# Nifurtimox Is Effective Against Neural Tumor Cells and Is Synergistic with Buthionine Sulfoximine

**DOI:** 10.1038/srep27458

**Published:** 2016-06-10

**Authors:** Michael Du, Linna Zhang, Kathleen A. Scorsone, Sarah E. Woodfield, Peter E. Zage

**Affiliations:** 1 Department of Pediatrics, Section of Hematology-Oncology, Texas Children’s Cancer and Hematology Centers, Baylor College of Medicine, Houston, TX 77030, USA

## Abstract

Children with aggressive neural tumors have poor survival rates and novel therapies are needed. Previous studies have identified nifurtimox and buthionine sulfoximine (BSO) as effective agents in children with neuroblastoma and medulloblastoma. We hypothesized that nifurtimox would be effective against other neural tumor cells and would be synergistic with BSO. We determined neural tumor cell viability before and after treatment with nifurtimox using MTT assays. Assays for DNA ladder formation and poly-ADP ribose polymerase (PARP) cleavage were performed to measure the induction of apoptosis after nifurtimox treatment. Inhibition of intracellular signaling was measured by Western blot analysis of treated and untreated cells. Tumor cells were then treated with combinations of nifurtimox and BSO and evaluated for viability using MTT assays. All neural tumor cell lines were sensitive to nifurtimox, and IC50 values ranged from approximately 20 to 210 μM. Nifurtimox treatment inhibited ERK phosphorylation and induced apoptosis in tumor cells. Furthermore, the combination of nifurtimox and BSO demonstrated significant synergistic efficacy in all tested cell lines. Additional preclinical and clinical studies of the combination of nifurtimox and BSO in patients with neural tumors are warranted.

Neural tumors, including tumors of both the central and peripheral nervous systems, are common in both adults and children and are often associated with aggressive disease and poor prognosis. Survivors of these neural tumors suffer from significant late effects from therapy. Neuroblastoma and medulloblastoma are among the most common forms of cancer seen in children, while glioblastoma is the most common and most aggressive malignant primary brain tumor that affects both adults and children. Patients with aggressive forms of these neural tumors have very poor outcomes, with limited responses to treatment and frequent relapses. While recently introduced reatment strategies, such as immunotherapy for upfront treatment of neuroblastoma and new molecular prognostication for medulloblastoma, have resulted in some improvement in patient outcomes, novel therapies continue to be sorely needed for the patients with these neural tumors.

Nifurtimox is a nitrofuran compound that has been used since the 1970’s as a primary form of therapy for Chagas’ disease, a parasitic infection caused by *Trypanosoma cruzi*^1^,^2^. Nifurtimox has been shown to act through production of reduced oxygen metabolites superoxide and hydrogen peroxide, for which the parasites have lower detoxification capacity^1^,^3^. Preclinical studies have also shown that nifurtimox treatment generates reactive oxygen species and inhibits neuroblastoma and medulloblastoma cell growth *in vitro* and *in vivo*^4^,^5^, and in early phase clinical trials, nifurtimox was well tolerated by children with relapsed neuroblastoma, with mild nausea, vomiting and anorexia being the most commonly observed side effects. Tumor responses were seen in patients treated with nifurtimox at a dose of 30 mg/kg/day both as a single agent and in combination with chemotherapy^6^.

Buthionine sulfoximine (BSO) is a selective inhibitor of γ-glutamylcysteine synthetase (γ-GCS), the rate limiting enzyme in glutathione (GSH) synthesis^7^. BSO has been shown to increase the efficacy of nifurtimox against *T. cruzi*^8^,^9^ and has also been shown to be an effective modulator of GSH-mediated chemoresistance by increasing the *in vitro* cytotoxicity of alkylating agents and radiation^10^,^11^,^12^,^13^,^14^. BSO has been tested in animal studies and in human phase I trials for adults with solid tumors, with documented clinical responses in patients with melanoma, ovarian carcinoma and small cell carcinoma of the lung treated with the combination of BSO and melphalan^15^,^16^. BSO was also shown to be effective against neuroblastoma tumor cells, both alone and in combination with melphalan^17^,^18^,^19^, and prior studies have reported that treatment of mice with human glioma and medulloblastoma xenograft tumors with BSO prior to melphalan resulted in increased survival^20^. Furthermore, a pilot clinical trial for treatment of children with recurrent neuroblastoma with BSO combined with melphalan demonstrated an 18% response rate^21^, suggesting a potential role for BSO in combination with other therapies for cancer patients.

Despite these prior studies, the efficacy of nifurtimox against other neural tumor cells has not been well characterized, and the efficacy of the combination of nifurtimox and BSO has not been fully explored in preclinical models of cancer. Based on the evidence for the potential efficacy of nifurtimox and BSO as single agents against neural tumors and the efficacy of the combination against *T. cruzi*, we hypothesized that nifurtimox would be effective against neural tumor cells alone and in combination with BSO.

## Materials and Methods

### Cells and culture conditions

The neuroblastoma cell lines used in this study have been previously utilized by our laboratory^22^,^23^, and were either purchased from American Type Culture Collection (ATCC, www.atcc.org) or were generously provided by Susan Cohn (The University of Chicago Children’s Hospital, Chicago, IL, USA), John Maris (Children’s Hospital of Philadelphia, Philadelphia, PA, USA), or the Children’s Oncology Group (COG) Cell Culture and Xenograft Repository (www.cogcell.org). Neural tumor cell lines U-87, U373, CHLA-02-ATRT and PFSK-1 were purchased from ATCC. Medulloblastoma cell lines Daoy and D283 were generously provided by Vidya Gopalakrishnan (The University of Texas MD Anderson Cancer Center, Houston, TX, USA). TC-71, 143B, MG-63, MMH-ES, RD-ES, SK-ES-1 and SK-N-MC cells were generously provided by Jason Yustein (Baylor College of Medicine, Houston, TX, USA). Cell lines were grown at 37 °C in 5% CO_2_ in appropriate media (Invitrogen, Carlsbad, CA) supplemented with 10% heat-inactivated fetal bovine serum (FBS) (Life Technologies, Grand Island, NY), L-glutamine, sodium pyruvate and non-essential amino acids (Sigma-Aldrich, St. Louis, MO). All cell lines were authenticated by DNA profiling prior to use.

### Therapeutic agents

Nifurtimox was generously provided by MetronomX, Inc. (Houston, TX). A 70 mM stock solution was generated in DMSO (Sigma) and stored at −20 °C. Nifurtimox was diluted in PBS to appropriate concentrations immediately before use. BSO (Sigma) was diluted in water (50 mg/mL) and stored at −20 °C. BSO was warmed and diluted in PBS to appropriate concentrations immediately prior to use.

### Cell viability assays

The viability of cells exposed to nifurtimox and BSO was determined using a modified methyl tetrazolium (MTT; Sigma) assay as previously described^22^,^23^. 0.5–1.0 × 10^4^ cells/well of exponentially growing cells were plated in 96-well plates. 24 hours later, nifurtimox or BSO was added to each well at specified concentrations. After 72, 96, or 120 hours of continuous drug exposure, 15 μL of 5 mg/ml MTT was added to each well and the plates were incubated for 4 hours at 37 °C. Medium was replaced with 150 μL of DMSO and the optical density (OD) was measured at 550 nm using a microplate spectrophotometer (Anthos Analytical, Durham, NC). Cell numbers were estimated from OD measurements in individual wells. Relative cell viability was calculated by subtracting the background OD of media alone and then dividing by the OD of control wells. Replicates of three wells were used for each drug concentration and assays were duplicated on separate days. IC50 values were derived using best-fit trendlines and values calculated using the relevant curve-fit equations.

For combination studies using nifurtimox and BSO, average cell viability for each drug alone was calculated and plotted against individual drug concentrations. Cells were then plated as above, treated with the specified concentrations of nifurtimox and BSO (with separate 96-well plates for each combination) for 72 hours, with cell viability determined as above and combination indices calculated using CalcuSyn v2.11 software (Biosoft, Cambridge, UK).

### Inhibition of Signaling Assays

Neural tumor cell lines were plated at approximately 80% confluency in 60 mm plates and allowed to adhere overnight. Plates were washed twice with PBS and incubated in media for three hours, followed by the addition of nifurtimox for 72 hours. Cells were washed with PBS and lysed with M-PER Mammalian Protein Extraction Reagent (Thermo Scientific, Rockford, IL) with protease inhibitor (Sigma) at the completion of each experiment. Lysates were centrifuged and supernatants were collected.

Protein concentration in each sample was measured using a protein assay dye reagent (Bio-Rad, Hercules, CA). 30–50 μg total denatured protein from each cell line was separated by sodium dodecyl sulfate-polyacrylamide gel electrophoresis (SDS-PAGE) and transferred to nitrocellulose membranes (Invitrogen, Carlsbad, CA) using standard techniques. Membranes were blocked in Odyssey blocking buffer (Li-Cor, Lincoln, NE) for 1 hour at room temperature and then incubated with primary antibodies to total ERK (#4695, 1:1000; Cell Signaling, Danvers, MA, USA), phosphorylated ERK (#4370, 1:1000; Cell Signaling), total MEK (#9126, 1:1000; Cell Signaling), phosphorylated MEK (#9154, 1:1000; Cell Signaling), β-actin (#A3853, 1:5000; Sigma), or vinculin (ab1290002, 1:2000; Abcam). All antibodies were diluted in Odyssey blocking buffer (Li-Cor) with 0.1% Tween-20. Bound primary antibodies were incubated for 1 hour at room temperature with IRDye800 conjugated affinity purified anti-rabbit or anti-mouse secondary antibody (1:2000; Rockland, Gilbertsville, PA) and the signal was visualized on an Odyssey infrared imaging system (Li-Cor).

### Apoptosis assays

Apoptosis was measured using assays for DNA ladder formation and poly-ADP ribose polymerase (PARP) cleavage. For DNA ladder formation assays, neural tumor cells were plated in 6-well plates and allowed to adhere overnight. Cells were then washed twice with PBS, followed by the addition of nifurtimox for 24 hours. Cells were collected and incubated in cell lysis buffer (100 mM Tris pH 8.0, 50 mM EDTA and 1% SDS (w/v)) for 10 minutes at room temperature. The mixture was vortexed with protein precipitation buffer (6 M NaCl) at room temperature followed by centrifugation. Genomic DNA was precipitated from the supernatant with isopropyl alcohol. After centrifugation, the remaining DNA precipitate was washed with 70% ethanol. The final DNA was dissolved in TE buffer (10 mM Tris-HCl, 1 mM EDTA) at 37 °C. A Nanodrop spectrophotometer (Thermo Scientific) was used to measure DNA concentrations. 5 μg of total genomic DNA was separated on a 1% agarose gel and photographed with a VersaDoc molecular imager (Bio-Rad).

For PARP cleavage assays, neural tumor cells were plated as above and exposed to nifurtimox for 72 hours. Treated and untreated control cells were washed and lysed as above and 30–50 μg total denatured protein from each cell line was separated by SDS-PAGE and transferred to nitrocellulose membranes as above. Membranes were blocked and incubated with primary antibodies to PARP1 (#9542, 1:500, Cell Signaling). After exposure to secondary antibodies as above, images were obtained using the Odyssey infared imaging system.

## Results

### Neural tumor cell line sensitivity to nifurtimox

**Figure 1:**
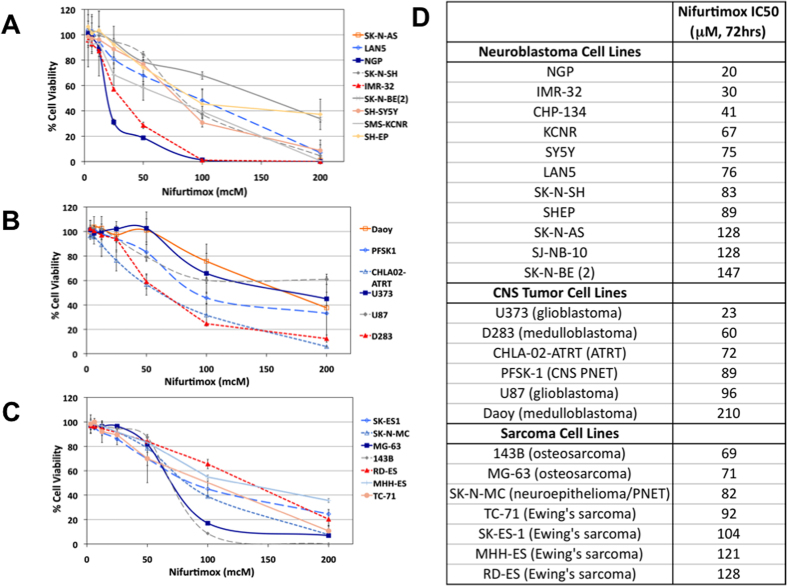
Effects of nifurtimox on tumor cell lines. (**A**) panel of neuroblastoma (**A**), CNS (**B**) and sarcoma (**C**) tumor cell lines were treated with increasing concentrations of nifurtimox for 72 hours and cell viability was determined by MTT assays. (**D**) IC50 values were calculated for each cell line.

In order to determine the efficacy of nifurtimox against neural tumor cells, a panel of established human tumor cell lines were tested for sensitivity *in vitro* to a range of concentrations of nifurtimox using MTT assays. IC50 values were calculated and ranged from 20 to 210 μM (Fig. 1). Increased exposure time resulted in slightly decreased IC50 values (Supplemental Fig. 1). NGP and U373 tumor cells were among the most sensitive cell lines to nifurtimox, while SK-N-BE(2) and Daoy cells were among the least sensitive (Fig. 1). Most cell lines had IC50 values <100 μM, including 8 of 11 tested neuroblastoma and 5 of 6 tested CNS tumor cell lines, suggesting that neural tumor cells were particularly sensitive to nifurtimox.

### Nifurtimox induces apoptosis and inhibits intracellular signaling in neural tumor cells

**Figure 2:**
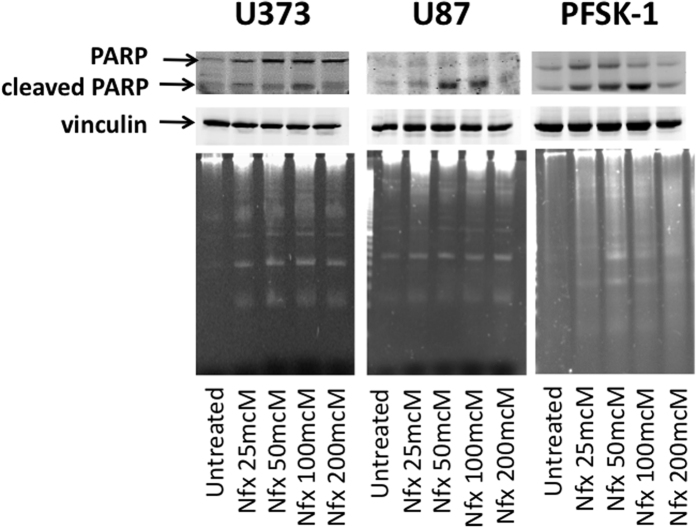
Nifurtimox treatment induces apoptosis in neural tumor cells. U373, U87 and PFSK-1 neural tumor cells were treated with nifurtimox at the concentrations shown and assayed by immunoblot for PARP cleavage (top) and by agarose gel electrophoresis for DNA ladder formation (bottom).

Based on the efficacy of nifurtimox as a single agent in our initial testing (Fig. 1) and the known ability of nifurtimox to penetrate the blood brain barrier^24^,^25^, we pursued further studies of nifurtimox in neural tumor cell lines. In order to determine the mechanism of decreased neural tumor cell viability after treatment with nifurtimox, we performed assays for DNA ladder formation and for cleavage of PARP to establish whether nifurtimox treatment induced apoptosis. DNA ladders and PARP cleavage were evident in tumor cells after treatment with nifurtimox (Fig. 2), suggesting that the decreased viability of tumor cells after nifurtimox treatment was secondary to induction of apoptosis.

**Figure 3:**
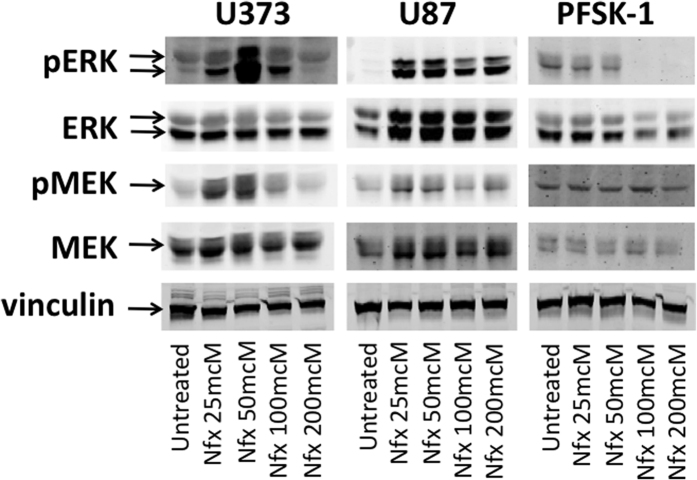
Nifurtimox treatment inhibits ERK phosphorylation in neural tumor cells. U373, U87 and PFSK-1 neural tumor cells were lysed at baseline (untreated) or after treatment with nifurtimox. Western blots were performed for total (ERK, MEK) and phosphorylated (pERK, pMEK) ERK and MEK. Vinculin was used as a protein loading control.

Activity of the RAS/MAPK signaling pathway has been linked to the pathogenesis of multiple cancers in both adults and children^26^. To determine whether treatment with nifurtimox resulted in inhibition of these downstream signaling pathways, U373, U87 and PFSK-1 neural tumor cell lines were harvested before and after nifurtimox treatment and Western blots for total and phosphorylated MEK and ERK were performed. Nifurtimox treatment resulted in loss of ERK phosphorylation in PFSK-1 cells and in increased phosphorylation of MEK and ERK in glioblastoma cell lines at low doses of nifurtimox, followed by significant reduction of MEK and ERK phosphorylation at doses above the determined IC50 values in these cell lines (Fig. 3).

### Neural tumor cell line sensitivity to BSO and to the combination of nifurtimox and BSO

**Figure 4:**
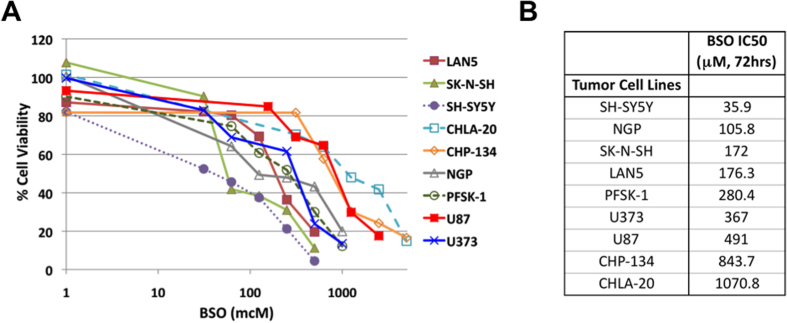
Effects of BSO on neural tumor cells. Neural tumor cells were treated with increasing concentrations of BSO for 72 hours and cell viability was determined by MTT assays (**A**). (**B**) IC50 values were calculated for each independent cell line.

BSO has demonstrated antitumor activity in a number of preclinical cancer models of neural tumors^17^,^18^,^19^,^20^. In order to confirm the efficacy of BSO in neural tumor cell lines, 6 neuroblastoma, 2 glioblastoma, and 1 PNET cell line were treated with increasing concentrations of BSO, and viability was determined using MTT assays. BSO was effective against neural tumor cell lines, with IC50 values ranging from 36 to 1071 μM (Fig. 4), consistent with previously reported IC50 values for BSO in other neuroblastoma cell lines^18^.

**Figure 5:**
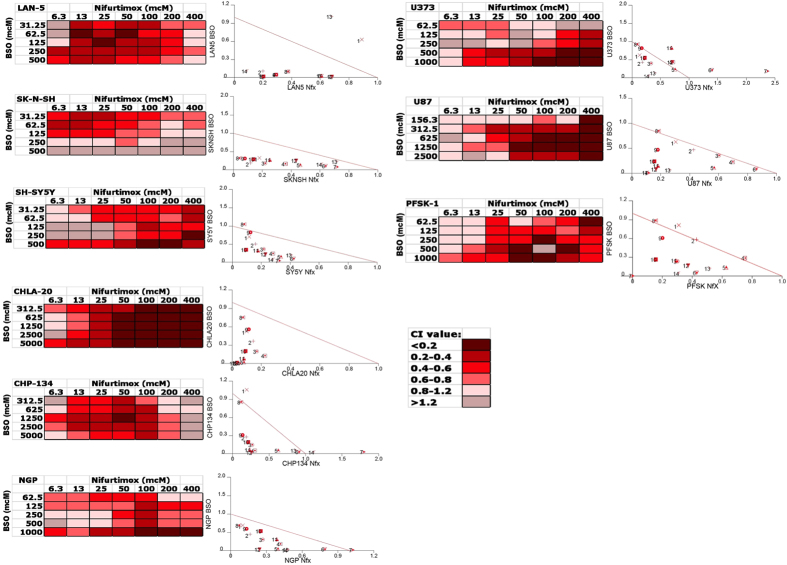
Combination indices for nifurtimox plus BSO in neural tumor cell lines. Combination indices (CIs) were calculated for each cell line and isobolograms were generated using all calculated CIs for each individual cell line (left). CIs were then color coded and plotted for each individual cell line (right).

In order to determine whether BSO could enhance the efficacy of nifurtimox against neural tumor cells, the panel of neural tumor cell lines were treated with the combination of nifurtimox and BSO over a range of concentrations. In each of the 9 tested cell lines, cell viability was dramatically reduced with the combination of nifurtimox and BSO, compared to either drug alone (Supplemental Fig. 2). Furthermore, calculated combination indices were consistently <1 for most tested dosing combinations in each of the 9 cell lines (Fig. 5), suggesting synergistic efficacy of nifurtimox and BSO in neural tumor cell lines.

## Discussion

New treatment strategies are clearly needed for patients with high-risk and recurrent neural tumors. We have demonstrated that the anti-trypanosomal agent nifurtimox is effective against neural tumor cells and is synergistic with the GSH inhibitor BSO. Nifurtimox treatment results in reduced ERK phosphorylation and in induction of apoptosis and our results suggest that nifurtimox alone and in combination with BSO may be effective for patients with all types of neural tumors, including neuroblastoma, glioblastoma and medulloblastoma.

Prior studies have demonstrated the efficacy of nifurtimox and BSO as single agents in selected neural tumor models^4^,^5^,^17^,^18^. Our results both confirm and expand these prior reports and demonstrate that nifurtimox is effective against a wide range of tumor cell types and that nifurtimox combined with BSO demonstrates synergistic drug efficacy. However, the specific disease subtypes and biologic prognostic features associated with responses to nifurtimox and BSO are currently unknown. We are currently investigating the associations with other biologic factors and signaling pathways with the responses of neural tumor cells to nifurtimox and BSO.

Oral nifurtimox has been used for long-term treatment of Chagas’ disease in adults at doses of 8 to 10 mg/kg/day and in children at 15 to 20 mg/kg/day^27^. When used to treat an acute *T. cruzi* infection, nifurtimox is administered for 90 days, but for chronic infection nifurtimox treatment continues for 120 days or longer^27^,^28^. Nifurtimox-related toxicities in adults primarily include nausea, vomiting, diarrhea, anorexia and weight loss along with irritability, sleep disorders and peripheral neuropathies^28^. During long-term treatment, additional secondary side-effects have included tremors, muscle weakness, mild paresthesia and polyneuritis^29^,^30^. Unlike adult patients, infants and young children with Chagas’ disease treated with nifurtimox only experience minor adverse events, including anorexia and diarrhea^31^,^32^. Toxicities observed in children with neuroblastoma treated with nifurtimox included anorexia, nausea, stomach pain, neuropathies, and seizures^6^, and pill compliance data showed that, on average, children treated with nifurtimox took 96.7% of their prescribed pills, showing that the treatment regimen could be easily followed. The maximum tolerated dose in this patient population was determined to be 30 mg/kg/day^6^.

We determined the efficacy of nifurtimox against a panel of neural tumor cell lines, with calculated IC50 values between 20 and 210 μM, similar to previously published results^4^,^5^. In healthy adults, serum levels of nifurtimox reached 2.6 μM after a single 15 mg/kg oral dose^33^,^34^, while peak serum levels in children with neuroblastoma treated with single agent nifurtimox at 30 mg/kd/day exceeded 1 mM^6^, suggesting that serum levels required for tumor cell effects in our *in vitro* models are clinically achievable in patients with neural tumors.

A key limitation of many potential novel therapies for both adults and children with intracranial tumors is the inability of these agents to penetrate the blood brain barrier (BBB). Nifurtimox has been previously shown to penetrate the BBB in preclinical models^24^,^25^, and nifurtimox has demonstrated efficacy in late stage African trypanosomiasis (African sleeping sickness), defined by the presence of active CNS infection^35^,^36^. It is unclear whether the penetrability of the BBB is similar in patients with infection compared to those with malignancies. However, nifurtimox has demonstrated efficacy in intracranial orthotopic models of neural tumors^4^,^5^, suggesting that nifurtimox may be able to penetrate the BBB and reach levels with antitumor efficacy in patients with glioblastoma and other intracranial tumors.

The mechanism(s) underlying the efficacy of nifurtimox in neural tumors is not fully understood. Nifurtimox is a nitroheterocyclic compound that undergoes cellular reduction to nitro anion free radicals, hydrogen peroxide, and superoxide free radicals, which generate intracellular reactive oxygen species (ROS), leading to antitrypanosomal efficacy^37^. Nifurtimox has also been shown to induce apoptosis via induction of ROS in the presence of catecholamines in neuroblastoma preclinical models^5^. However, the mechanims of efficacy in tumor cells in the absence of catecholamines is not clear.

Glutathione (GSH) is a ubiquitous intracellular tri-peptide that acts as a key intracellular antioxidant to minimize damage to important cellular components caused by ROS such as free radicals, peroxides, and heavy metals. The antitumor activity of many chemotherapy agents depends upon the induction of ROS, and inhibition of GSH has been shown to increase the efficacy of chemotherapy^38^. As a selective inhibitor of γ-GCS, the rate limiting enzyme in GSH synthesis, BSO has been shown to deplete GSH both *in vitro* and *in vivo*^10^,^11^,^18^, and BSO-mediated depletion of GSH renders cells susceptible to alkylating agent-mediated DNA damage, anthracycline-mediated lipid peroxidation of the cellular membranes, and ionizing radiation^16^,^17^,^18^. Indeed, BSO has been shown to be synergistic with multiple chemotherapy agents^10^,^11^,^38^,^39^,^40^,^41^,^42^,^43^,^44^,^45^.

BSO has been shown to be cytotoxic for neuroblastoma cell lines *in vitro* due to increased ROS^18^,^19^, and BSO has been shown to enhance the efficacy of nifurtimox in a murine model of Chagas’ Disease^9^, suggesting a likely mechanism for our observed synergistic efficacy with nifurtimox. Our determined IC50 values were similar to previously published values in similar cell lines^18^ and were also within the range of serum BSO levels seen in adults and children treated with BSO^21^,^46^, suggesting that serum levels of BSO in our *in vitro* models are clinically achievable in patients with neural tumors.

We have demonstrated that nifurtimox is effective against neural tumor cells and is synergistic with BSO. All tested neural and non-neural tumor cell lines were sensitive to nifurtimox, and in neural tumor cells, nifurtimox treatment inhibited ERK phosphorylation and induced apoptosis. The combination of nifurtimox and BSO demonstrated synergistic efficacy in tested cell lines, and further preclinical studies of the combination of nifurtimox and BSO are warranted. Furthermore, the efficacy of this combination against neural tumor cells provides strong biological and clinical rationale for clinical testing of nifurtimox and BSO in patients with additional types of neural tumors.

## Additional Information

**How to cite this article**: Du, M. *et al*. Nifurtimox Is Effective Against Neural Tumor Cells and Is Synergistic with Buthionine Sulfoximine. *Sci. Rep.* **6**, 27458; doi: 10.1038/srep27458 (2016).

## Supplementary Material

Supplementary Information
