# Supplementary material for: Nifurtimox Is Effective Against Neural Tumor Cells and Is Synergistic with Buthionine Sulfoximine
Source: Sci Rep. 2016 Jun 10;6:27458. doi: 10.1038/srep27458 (PMC4901277; doi:10.1038/srep27458)
Supplement: Supplementary Information [file srep27458-s1.pdf]

Nifurtimox Is Effective Against Neural Tumor Cells and Is Synergistic with Buthionine Sulfoximine

Michael Du; Linna Zhang, MD, MS; Kathleen A. Scorsone, PhD; Sarah E. Woodfield, PhD; Peter E. Zage, MD, PhD*
Department of Pediatrics, Section of Hematology-Oncology, Texas Children's Cancer and Hematology Centers, Baylor College of Medicine, Houston, TX 77030

*Address correspondence/requests for reprints to:
Peter Zage, MD, PhD
University of California San Diego School of Medicine
Moores Cancer Center, Room #5311
3855 Health Sciences Drive, MC 0815
La Jolla, CA 92093-0815
Tel: 858-534-6494
email: pzage@ucsd.edu

Supplemental Figure S1 *Effects of exposure time on tumor cell line responses to nifurtimox*. A panel of neuroblastoma tumor cell lines were treated with increasing concentrations of nifurtimox for 72 or 96 hours, and cell viability was determined by MTT assays.

Supplemental Figure S2 *Efficacy of nifurtimox combined with BSO in neural tumor cell lines.* Neural tumor cells were treated with increasing concentrations of nifurtimox combined with BSO for 72 hours, and cell viability was determined by MTT assays. Percent cell death was calculated for each cell line with each individual drug dose combination.

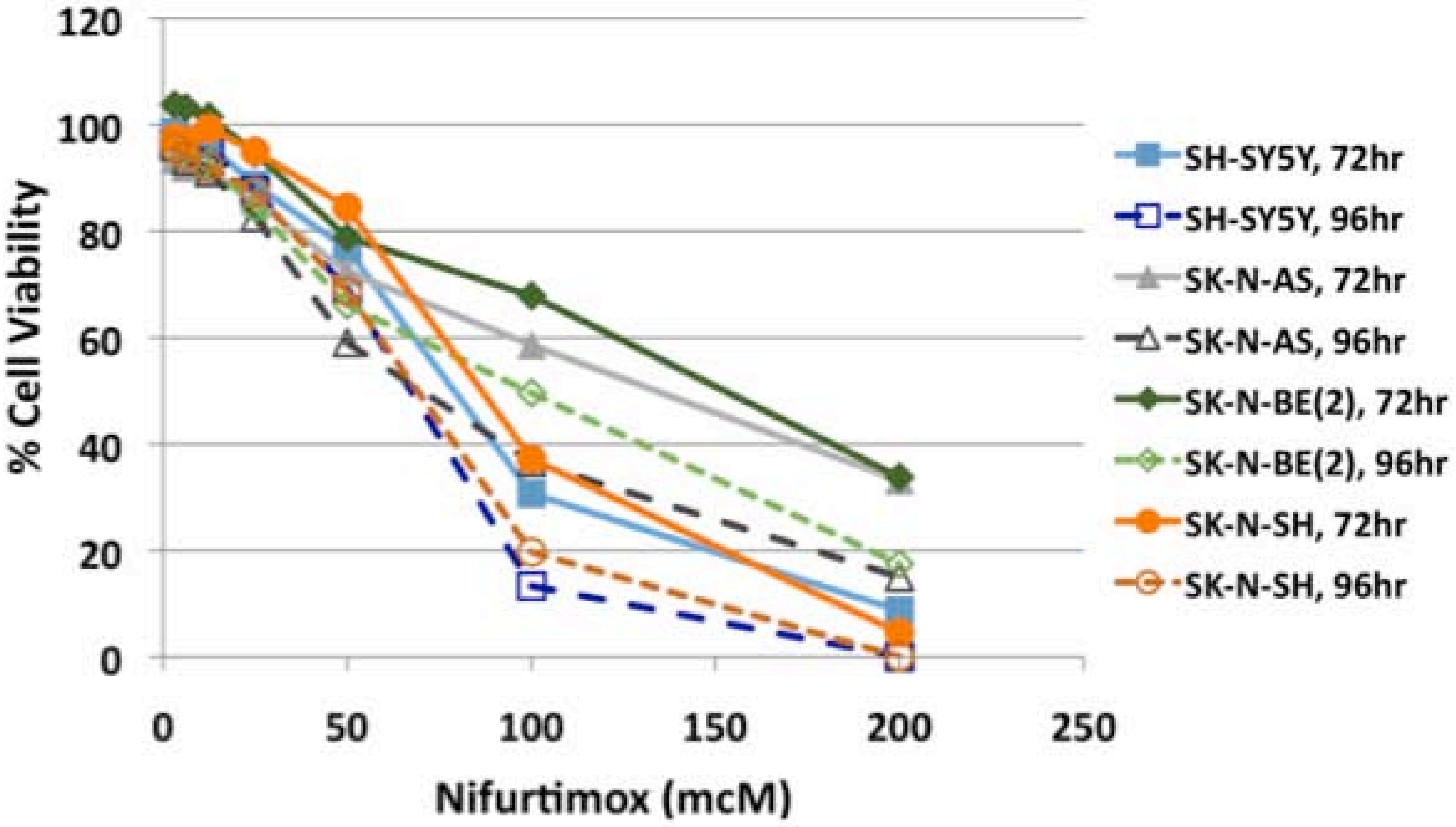

120
100
80
60
40
20
0
% Cell Viability
0
50
100
150
200
250
Nifurtimox (mcM)
SH-SY5Y, 72hr
SH-SY5Y, 96hr
SK-N-AS, 72hr
SK-N-AS, 96hr
SK-N-BE(2), 72hr
SK-N-BE(2), 96hr
SK-N-SH, 72hr
SK-N-SH, 96hr

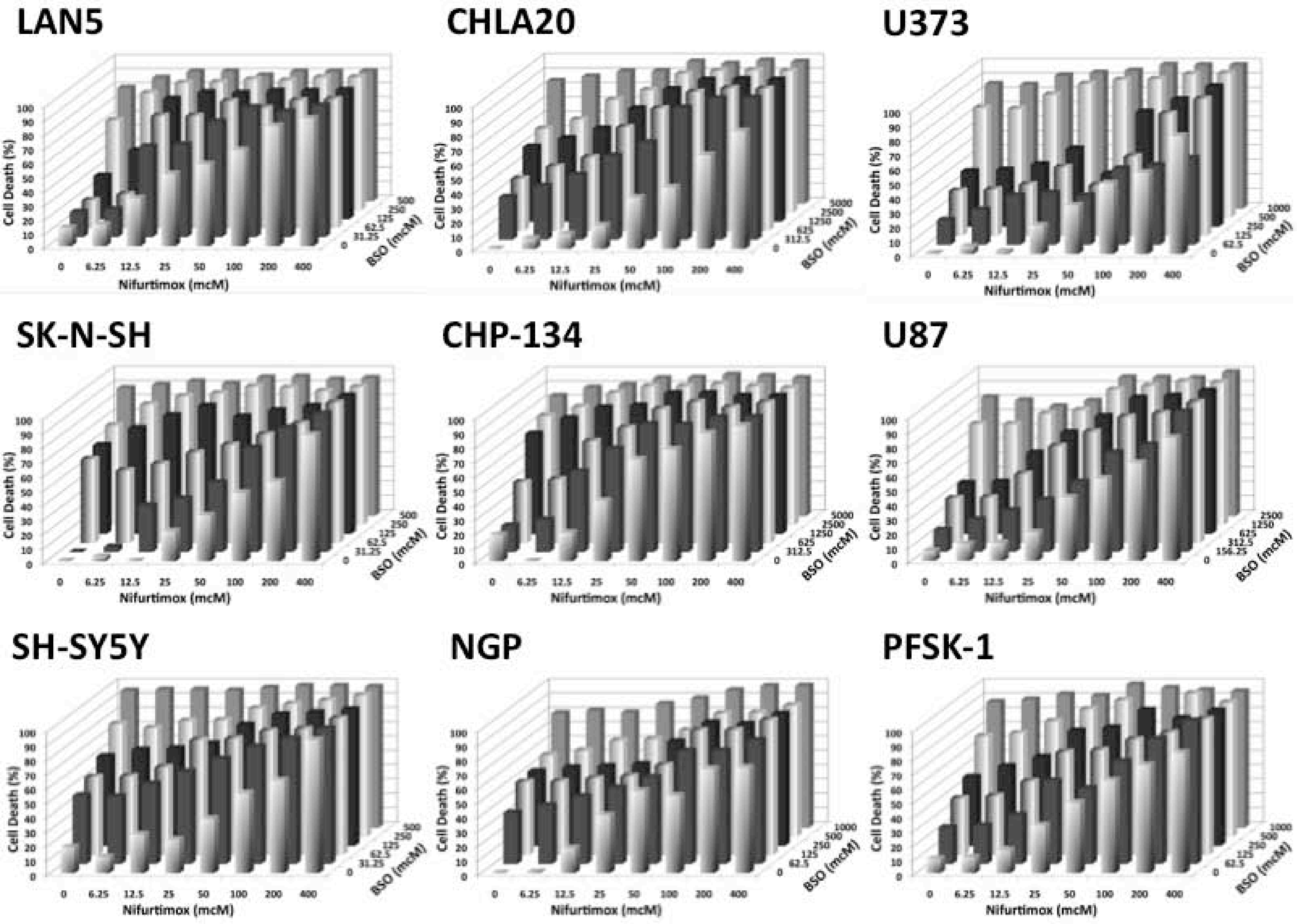

LAN5
CHLA20
U373
SK-N-SH
CHP-134
U87
SH-SY5Y
NGP
PFSK-1
Cell Death (%)
Nifurtimox (mcM)
BSO (mcM)
0
6.25
12.5
25
50
100
200
400
